# Vascular Endothelial Dysfunction in Inflammatory Bowel Diseases: Pharmacological and Nonpharmacological Targets

**DOI:** 10.1155/2018/2568569

**Published:** 2018-04-12

**Authors:** Antonietta Gerarda Gravina, Marcello Dallio, Mario Masarone, Valerio Rosato, Andrea Aglitti, Marcello Persico, Carmelina Loguercio, Alessandro Federico

**Affiliations:** ^1^Department of Precision Medicine, University of Campania “Luigi Vanvitelli”, Via Pansini 5, 80131 Naples, Italy; ^2^Department of Medicine and Surgery, University of Salerno, Via Salvador Allende, 84081 Baronissi, Salerno, Italy

## Abstract

Inflammatory bowel diseases, including Crohn's disease and ulcerative colitis, are chronic inflammatory conditions involving primarily the gastrointestinal tract. However, they may be also associated with systemic manifestations and comorbidities. The relationship between chronic inflammation and endothelial dysfunction has been extensively demonstrated. Mucosal immunity and gastrointestinal physiology are modified in inflammatory bowel diseases, and these modifications are mainly sustained by alterations of endothelial function. The key elements involved in this process are cytokines, inflammatory cells, growth factors, nitric oxide, endothelial adhesion molecules, and coagulation cascade factors. In this review, we discuss available data in literature concerning endothelial dysfunction in patients affected by inflammatory bowel disease and we focus our attention on both pharmacological and nonpharmacological therapeutic targets.

## 1. Introduction

Inflammatory bowel diseases (IBD) are chronic inflammatory pathologies that primarily involve the gastrointestinal tract associated with a combination of environmental, genetic, and immunological pathogenetic factors. The consequence of this “worsening cooperation” is an uncontrolled immune response against self-antigen of the intestine, which acts as a trigger in genetically predisposed individuals to disease development [1]. Crohn's disease (CD) may occur in any region of the gastrointestinal tract involving, in most of the cases, the ileum and colon with a discontinuous, transmural, and granulomatous inflammation pattern, whereas ulcerative colitis (UC) only affects the colon and rectum and is restricted to the mucosal layer of the intestine which appears with a continuous and exudative inflammation pattern [2, 3]. Several studies have reported that the prevalence of cardiovascular risk factors such as obesity, dyslipidemia, diabetes, and hypertension is lower among subjects affected by IBD in comparison to the general population [1, 4, 5]. In accordance with this observation, we would expect a lower cardiovascular mortality and morbidity in IBD patients. However, on the contrary, cardiovascular disease incidence in patients with IBD seems to be increased [6]. It can therefore be hypothesized that there are other factors that play an important role in cardiovascular disease development in these subjects, such as chronic inflammation [1].

In chronic systemic inflammation diseases, the inflammation affects the arterial properties and causes both endothelial dysfunction and an increase of arterial stiffness. A relationship between increased arterial stiffness and inflammatory disorders has been described in a lot of inflammatory diseases including systemic vasculitis [7], rheumatoid arthritis [8], and systemic lupus erythematosus [9]. In this review, we analyze the relationship between inflammatory bowel disease inflammation and endothelial dysfunction in order to predict the possible role of this inflammation in cardiovascular disease development. Moreover, we focus our attention on the possible pharmacological and nonpharmacological therapeutic targets oriented to interrupt this dangerous link in order to reduce the cardiovascular morbidity and mortality in this category of patients.

## 2. Main Text

### 2.1. Arterial Stiffness in Chronic Inflammatory Diseases

Several studies reported that arterial stiffness and endothelial function could be considered as markers of subclinical inflammation-associated organ damage [1, 10]. However, a small number of studies evaluated both endothelial function and arterial stiffness in subjects with IBD. Laurent et al. [11] in an expert consensus document described the gold standard procedure in order to assess regional arterial stiffness in daily practice and highlighted the direct relationship between arterial stiffness and the pulse wave velocity (PWV) measurement. PWV is measured by pressure waveforms obtained transcutaneously in correspondence to the right common carotid artery and the right femoral artery (carotid-femoral PWV). PWV is calculated by dividing the distance between two detection points for the time necessary to cover it. An increased carotid-femoral PWV is considered both a marker of target organ damage and a cardiovascular risk factor [1]. The relationship between arterial stiffness, PWV, and inflammation has been reported in patients with chronic inflammatory diseases, such as systemic vasculitis, and rheumatoid arthritis, and patients with increased concentrations of high-sensitivity C-reactive protein (hsRCP). Pietri et al. [12] reported a positive correlation between PWV, direct marker of arterial stiffness, and hsRCP, independently from blood pressure, in patients with untreated primary hypertension, as well as in normotensive individuals. Yasmin et al. [13] also reported the same data about healthy individuals. Endothelial dysfunction could be considered a possible mechanism linking inflammation and arterial stiffness. Inflammation may induce structural changes in the arterial wall, by altering the balance between elastin breakdown and synthesis. Indeed, several elastolytic enzymes, including matrix metalloproteinase-9, are known to be upregulated by inflammatory cytokines [13, 14]. Increased arterial stiffness in patients with inflammatory diseases could be reversible, since in patients with rheumatoid arthritis treated with drugs against tumor necrosis factor- (TNF-) *α*, some authors observed a reduced PWV comparable to that obtained by healthy individuals [8]. PWV has increased in patients with IBD without differences between CD and UC patients [15–19]. An increased functional and structural arterial stiffening was described in inflammatory diseases. These structural or functional changes are supported by endothelial dysfunction (Table 1).

### 2.2. Endothelial Dysfunction and IBD

Endothelial dysfunction is “an imbalance between vasodilating and vasoconstricting substances produced by (or acting on) endothelial cells” [20, 21]. Endothelial dysfunction is characterized by upregulation of cellular adhesion molecules, compromised barrier function, increased leukocyte diapedesis, and increased vascular smooth muscle tone. These phenomena are related to an impaired production of vasodilator substances such as nitric oxide (NO) as well as an increase of vasoconstrictor substances including endothelin that determine the appearance of a prothrombotic state [22].

Several authors demonstrated that sex and age could influence the endothelial function. Ciccone et al. showed in 2013 that endothelial dysfunction has worsened with advancing age and that it occurs earlier in males in comparison with women. In healthy men under 40 years, endothelial function seems to be preserved and after this phase of life, it seems to be worse; in healthy women, it seems to be preserved up to 50 years and decline thereafter [23].

Endothelial dysfunction has been widely demonstrated to be the first step in the development of atherosclerosis. The consequent alteration of the vasodilation due to endothelial dysfunction is considered as a cumulative result of the dangerous actions sustained by all the atherogenic factors. Indeed, some studies have shown that endothelial dysfunction is an independent risk factor for cardiovascular disease development [24, 25]. Endothelial function seems to be compromised in patients with IBD. Garolla et al. [26] demonstrated that the number of circulating endothelial precursor cells (EPCs), which are considered markers of both endothelial reparation and vascular healing, was significantly reduced in patients with IBD compared with healthy controls. Moreover, they also demonstrated that apoptotic endothelial precursor cells were higher in patients with IBD than in healthy controls. Finally, they hypothesized that in IBD patients, apoptosis contributes to the reduction of circulating EPC number and also influencing their ability to proliferate. This condition may represent a risk factor for cardiovascular disease and endothelial dysfunction in these patients [1].

**Figure 1:**
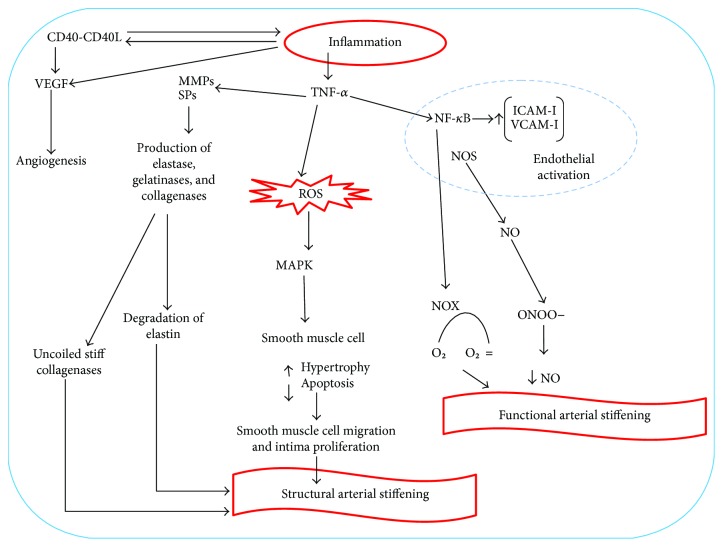
Mechanisms of inflammation-derived endothelial dysfunction. The CD40-CD40L pathway stimulates mucosal inflammation and causes increased production of proinflammatory cytokines, such as interleukin- (IL-) 8, chemokines, and cell adhesion molecules, and causes angiogenesis-stimulating intestinal fibroblasts to release angiogenic cytokines.

Endothelial function is ensured thanks to the maintenance of a balance among different elements such as NO, endothelin 1, von Willebrand factor (vWF), and cellular adhesion molecule (CAM) superfamily [20, 27]. Inflammation leads to structural and functional changes in the vascular endothelium and its activation. These changes initially include increased leukocyte adhesiveness, leukocyte diapedesis, vascular smooth muscle tone, and procoagulant activity [20, 22, 28]. The interactions between integrins and chemokine receptors with endothelial and mucosal ligands promote activation of endothelial cells [20, 29, 30]. The recruitment of leukocytes happens thanks to the endothelial expression of CAMs and chemokines [20, 31, 32]. The recruitment of leukocytes is mainly mediated by a link between leukocyte CD11a/CD18 and ICAM-1 in the gut or by a link between *α*4-*β*1 or *α*4-b7, VCAM-1, and mucosal addressin cell adhesion molecule-1 (MAdCAM-1) [20, 29]. Microvascular expression of ICAM-1, VCAM-1, and MAdCAM-1 is upregulated in patients with IBD [20, 31, 33]. MAdCAM-1 interacts with *α*4*β*7 integrins on the surface of a subset of naive CD4+ T-cells; therefore, an increase of MAdCAM-1 expression intensifies the recruitment of *α*4 integrin-expressing leukocytes [34–36]. Several inflammatory mediators, such RCP, are known to influence vascular functions. Wang et al. [37] demonstrated that RCP acts on vascular smooth muscle cells upregulating the angiotensin type I receptor and stimulating the migration and proliferation of smooth muscle cells, inducing, moreover, an increase in the production of reactive oxygen species (ROS). Pasceri et al. [38] demonstrated that RCP induces the secretion of some chemokines, adhesion molecules, and E-selectin from the endothelial cells, whereas Venugopal et al. [39] demonstrated that RCP decreases NOS expression. In contrast to these authors, Clapp et al. [40] in 2005 showed that RCP increases NO in a blood vessel cell model in vitro. However, further investigations are needed to establish if RCP is able to alter endothelial function, either favorably or unfavorably. Other inflammatory mediators implicated in vascular dynamics are IL-1, TNF-*α*, NO, vascular endothelial growth factor (VEGF), CD40-CD40 ligand, and IL-6, which are upregulated in IBD [41–45]. Increased levels of proinflammatory cytokines, such as IL-1 and TNF-*α*, and oxidative stress products are responsible for some structural changes in the muscle cells of the vascular walls because they induce an increase of the expression of matrix metalloproteinases and serine proteinases with subsequent degradation of elastin and collagen. Muscle cells of the vascular wall express osteoblast markers and are able to take up phosphate and produce bioapatite. This process produces wall calcifications and reduces vessel elasticity [1, 46]. Inflammatory cells, such as macrophages, lymphocytes, mast cells, and fibroblasts, produce angiogenic factors and promote pathological angiogenesis in inflammatory tissues [20, 33, 47]. VEGF, fibroblast growth factor, and TNF-*α* upregulation are stimulated by hypoxia in the inflamed area, with the successive production of vessels [20, 47]. A significant increase in endothelial CD40 expression is also reported in patients with active IBD and it results in increased recruitment of leukocytes expressing CD40L and also of platelets [20, 34]. CD40 has been found in atherosclerotic plaques and is overexpressed in both intestinal mucosa and circulating platelets of IBD patients. The CD40-CD40L pathway stimulates mucosal inflammation and causes increased production of proinflammatory cytokines, such as IL-8, chemokines, and cell adhesion molecules, and causes angiogenesis-stimulating intestinal fibroblasts to release angiogenic cytokines [20, 42] (Figure 1). CD40 binding stimulates the production of TNF-*α*, which increases CD40 expression [20, 43]. These mechanisms are the basis of structural changes in the vessel wall, including capillary and venule remodeling and proliferation of endothelial cells.

NO is a mediator that plays a critical role in vascular homeostasis. It is generated from conversion of L-arginine to citrulline by NOS isoforms. Mammals have three isoforms of NOS, two of them are constitutive: endothelial NOS (eNOS) and neuronal NOS (nNOS); the other one is produced in response to inflammatory stimuli (inflammatory cytokines): inducible NOS (iNOS) [48]. NO increases the concentration of cyclic guanosine monophosphate, which has a vasodilation effect, inhibits the expression of cytokines, chemokines, and leukocyte adhesion substances, inhibits blood platelet adhesion and aggregation, and limits the proliferation of smooth muscle cells in the vascular wall. Arginase is an enzyme that acts in the opposite way to NOS and its expression is increased in IBD patients. For this reason, there is a decreased NO production in patients with IBD [20, 33, 49, 50]. eNOS-derived NO is a radical scavenger able to absorb O_2_ and generate the potent oxidant peroxynitrite (NO_3_^−^). TNF-*α* expression is increased in patients with IBD, binds TNF receptor and leads to diminished eNOS protein expression, and suppresses eNOS activity. Therefore, there is a low NO availability [20, 22] that consequently a vasoconstriction occurs, because of smooth muscle cell relaxation reduction. This mechanism is responsible for functional increase of arterial stiffness observed among subjects affected by chronic inflammation [1]. The levels of asymmetric dimethylarginine (ADMA) plasma, an endogenous eNOS inhibitor, are inversely correlated with NO plasma levels, and it is elevated in numerous diseases associated with cardiovascular risk; indeed, high ADMA levels are also associated with an increased cardiovascular risk [22, 51, 52]. Chronic inflammatory diseases are generally associated with increased oxidative stress. Proinflammatory cytokines, including TNF-*α*, are mainly responsible for the increase of ROS production in inflammatory diseases. TNF-*α* increases activity of NADPH oxidases (NOX), which catalyze the transfer of electrons to molecular oxygen in order to generate superoxide by neutrophils and endothelial cells [22, 53, 54]. Superoxide reacts with NO to produce peroxynitrite, thereby decreasing NO bioavailability. In addition to its production by NOS and metabolism by ADMA, NO bioavailability is also modulated by ROS [55]. Superoxide and other ROS are capable to increase the activity of nuclear factor- (NF-) *κ*B, a critical step in the transformation of endothelial cells in “activated cells” characterized, in part, by an increase of surface expression of CAMs [22, 56, 57]. NF-*κ*B activation may also stimulate NOX expression, further enhancing ROS production in the endothelium and regenerating the destructive loop of inflammation and oxidative stress [22, 58]. ROS produced in the inflamed area inhibit cleavage of vWF molecules and it may cause microvascular thrombosis in patients with IBD [27, 30, 59]. An increase of plasminogen activator inhibitor type 1 (PAI-1) and reduction of tissue-type plasminogen activator (t-PA) and urokinase-type plasminogen activator (u-PA) have been found in mesenteric vascular walls of patients with IBD [60, 61]. It means that the coagulation process is deeply altered in IBD. ROS production induces smooth muscle cell hypertrophy and intima proliferation through the activation of protein kinases activated by the mitogen (MAPk) pathway (Table 2). The endothelial dysfunction could be diagnosed through two main methods: physical and biochemical method. The first one is based on assessing vasodilation in large arteries in response to increased flow and receptor stimulation, mainly acetylcholine [20]. The most sensitive and widely used is the flow-mediated vasodilatation (FMD), but it is less sensitive in detecting early changes of the endothelium function, similarly to each physical method. Several studies demonstrated a decrease of FMD in IBD patients with active diseases but no changes in the carotid intima-media thickness compared to healthy control (c-IMT) [20, 62]. Roifman et al. [41] demonstrated lower pulse arterial tonometry (PAT) values in patients with IBD compared to healthy control. Theocharidou et al. [63] reported an increase of c-IMT in patients with IBD; however, they did not find any correlation with the activity of the diseases. c-IMT is the main early vascular wall morphological change preceding plaque formation [64–66]. Although some studies [62, 67, 68] did not find any difference in c-IMT values between patients with IBD and controls, other studies [63–65] identified a higher c-IMT in the IBD group than in the control group, even if patients and controls did not show higher cardiovascular risk factors [66] (Table 3).

Biochemical methods are based on the assessment of the synthesis of compounds produced by both normal and damaged endothelium. Different studies evaluated these markers; however, the outcomes are difficult to interpret. Some studies reported an increase of the levels of VEGF, ICAM-1, and E-selectin in the serum of patients with IBD [69, 70]. Magro et al. [71] demonstrated lower levels of angiogenetic factors (P-selectin, E-selectin, VCAM, ICAM, and VEGF) in serum of patients with inactive CD than of controls, thus suggesting a dysfunction of angiogenic process and wound repair. Other reliable biochemical methods have been described in the diagnosis of endothelial dysfunction by using biochemical parameters (Table 3).

### 2.3. Cardiovascular Risk and IBD

Chronic inflammatory diseases are associated with accelerated atherosclerosis and increased risk of cardiovascular diseases (CVD) with increased cardiovascular morbidity and mortality compared to the general population [5, 22, 72, 73]. The risk for CVD is controversial in patients with IBD, since different studies highlighted an increased risk for CVD [5, 20, 74, 75], whereas others demonstrated lack of evidence for an increased risk of mortality due to CVD (Table 4) [6, 20, 76, 77]. Ozturk et al. [78] suggested that patients with IBD without classic cardiovascular risk factors have a higher risk for endothelial dysfunction and atherosclerosis. Ciccone et al. demonstrated a strong correlation among body mass index (BMI), inflammation indices, RCP, erythrocyte sedimentation rate (ESR), and physical parameters of endothelial dysfunction, c-IMT, and FMD, in obese children. Because the presence of these factors is strongly related to endothelial function and to the development of atherosclerosis, the authors themselves stated that atherosclerosis could begin very early in life, during childhood, and the same author showed that the worsening of endothelial function was related to age [79]. IBD in active phase was related to enhanced risks of worse CVD outcome; on the other hand, no risk increase was found in remission compared to the control group in a large number of studies [5, 80]. Inflammatory mediators, such as RCP, TNF-*α*, IL-6, IL-18, and CD40L, are involved in the pathogenesis of inflammation and atherosclerosis [81, 82]. Endothelial dysfunction represents a very important pathogenetic key step in the initiation and maintenance of atherosclerosis in the general population and may be a marker for a future risk of cardiovascular events [74]. Inflammatory process underlies endothelial dysfunction and atherosclerosis pathogenesis; therefore, mechanisms linking systemic inflammatory diseases and atherosclerosis may be better understood with the analysis of the endothelium. Multiple factors, including circulating inflammatory cytokines, TNF-*α*, ROS, oxidized low-density lipoprotein (LDL), and traditional risk factors, activate, directly and indirectly, endothelial cells leading to impaired vascular relaxation, increased leukocyte adhesion, increased endothelial permeability, and generation of a prothrombotic state. The presence of endothelial dysfunction has been further considered in active phases of the diseases. However, this observation has been reached by comparing the active phase of the diseases to the control group. There is no certain data about a direct comparison of active and remission phases of the diseases [80], though IBD implies an increased cardiovascular risk [60, 83, 84] and the entity of the risk directly correlates with disease activity in a lot of studies. In these studies, the induction of the remission is able to reverse endothelial dysfunction in IBD, achieving a level similar to non-IBD subjects. This evidence allows to hypothesize that an adequate medical management of IBD may be able to reverse the increased cardiovascular risk characterizing active disease [85, 86, 87]. Adequate disease management would therefore be important already in childhood; as shown by Ciccone et al., atherosclerosis is a process that can begin in childhood; we should always try to manage patients with IBD well to keep the level of these atherosclerotic factors as always low, because several studies demonstrated presence of a lot of atherosclerotic markers in children affected by IBD [79].

Several studies have demonstrated an increased risk of cardiovascular disease in patients with IBD; however, regarding mortality risk, the evidences are less clear. Kristensen et al. [85] did not find an increased risk for CVD in patients with IBD without classic CVD risk factors after a 2-year follow-up. Singh et al. [88], in a meta-analysis of about 33 observational studies, showed a higher risk for ischemic heart disease and arterial thromboembolism in patients with IBD, but the increased risk for cardiovascular mortality was not observed. Fumery et al. [89], in a meta-analysis of 9 studies, demonstrated that patients with IBD had a significant increase in the risk of cardiovascular morbidity, particularly in women; however, in this paper, the mortality was not addressed. Kristensen et al. [85], in a cohort study, demonstrated an increased risk of myocardial infarction in patients with IBD during the active phase, whereas no risk was observed in remission. Dorn et al. [6], in a 2007 meta-analysis of 11 studies, failed to demonstrate an increased risk of cardiovascular mortality in patients with IBD. Consequently, they concluded that IBD was not associated with a higher incidence of cardiovascular disease. This last cited meta-analysis had numerous drawbacks [6, 41]. It is important to emphasize that some patients with CD are tobacco smokers; indeed, tobacco may also contribute to worsen endothelial damage [90]. These findings indicate that prospective studies are needed to determine the actual risks for CVD in patients with IBD.

### 2.4. Therapy: Pharmacological and Nonpharmacological Targets

IBD development consists of active and remission periods, and the aim of the therapy is to suppress the active phases. The endothelial dysfunction that underlies the increased cardiovascular risk in these patients is sustained by inflammation and oxidative stress. Therefore, the reduction of these two factors is associated with a reduction of endothelial dysfunction. In chronic inflammatory diseases, there are two types of treatments that can reduce the mediators of inflammation and oxidative stress. The first one is the classical drug therapy that is used to reduce the inflammation associated with the disease. Another therapy, widely used in clinical practice in patients with IBD, is anti TNF-*α* therapy, infliximab, or biosimilars. TNF-*α* is a very important cytokine in IBD, whose overexpression appears to be a common element in IBD pathogenesis. TNF-*α* is a cytokine involved in the pathogenesis and progression of atherosclerosis [91]. This cytokine seems to have a key role, as previously described, in endothelial dysfunction; indeed, intravascular administration of recombinant TNF-*α*, in both humans and experimental animals, leads to a reduction in endothelium-dependent relaxation in vitro and in vivo [25, 92]. Some authors showed that treatment with infliximab, in rheumatoid arthritis, improves endothelial dysfunction since it improves FMD, even if all the treatments used for rheumatoid arthritis tend to improve FMD. Therefore, further studies are needed to better understand the best therapy to be used in order to reduce endothelial dysfunction among these patients [93]. Mäki-Petäjä et al. [8] demonstrated that anti-TNF-*α* therapy ameliorated aortic stiffness, evaluated by PWV, compared to healthy subjects in patients with rheumatoid arthritis. With respect to IBD, there is a lack of data in literature about the effects of anti-TNF-*α* and CVD in IBD patients and about endothelial dysfunction and the role of anti-TNF-*α* regarding this field. Schinzari et al. [25] demonstrated that endothelial dysfunction is beneficially affected by intravascular TNF-*α* neutralization in patients with CD. Danese et al. [94] reported that anti-TNF-*α* can reduce thrombus formation and adhesion to the endothelium by interfering with the CD40/CD40L pathway.

The second treatment is a nonpharmacological therapeutic approach, because it is based on substances with antioxidant properties among which there are natural and synthetic antioxidants. Several authors have reported the involvement of oxidative stress in the pathogenesis of IBD and consequently the presence of ROS, such as anion peroxide and hydrogen peroxide, into the mucosa of patients with IBD and in experimental colitis models. Oxidative stress also underlies endothelial dysfunction, as previously mentioned; for this reason, it can be deduced that by reducing endothelial dysfunction through the use of antioxidants, it should also improve.

Natural antioxidants contain a wide variety of compounds, mainly phenol and polyphenols, flavonoids, carotenoids, steroids, and thiol. They can prevent cell vascular damage, thus reducing the risk of chronic diseases [48, 95]. Among the natural antioxidant compounds studied in the prevention of vascular damage are vitamin E, vitamin C, goji berries, thymus extracts, rosemary, green tea, and garlic, as reported in Table 5. Vitamin E prevents ROS overproduction, improving the release of prostacyclin, a powerful vasodilator and inhibitor of platelet aggregation. Vitamin E supplementation has been proposed in the diet in order to reduce cardiovascular risk [48, 96, 97]. Vitamin C prevents damage from lipid peroxidation by free oxygen radicals [48, 98–100]. Goji berries increase endogenous antioxidant power; they are able to increase the activity of antioxidant enzymes such as superoxide dismutase (SOD), catalase (CAT), and glutathione peroxidase (GSH-Px) [48, 101, 102]. Some authors have reported that the treatment with SOD, an enzyme that converts the superoxide anion into hydrogen peroxide, has a healthy effect in both the prevention of experimental colitis and its treatment [103, 104]. Seguí et al. [105, 106] demonstrated that treatment with SOD, in a model of experimental colitis, improved the severity of intestinal damage from both a macroscopic and a microscopic point of view. Seguí et al. demonstrated that SOD induced a reduction in the expression of adhesion molecules, such as VCAM-1, ICAM-1, and MAdCAM-1, and therefore as a consequence, the recruitment of leukocytes at the site of inflammation. Thymus extracts have a free radical scavenging activity [107, 108]. Green tea has an anti-inflammatory activity, reducing both the expression of both cyclooxygenases, the constituent one (COX-1) and the inducible one (COX-2), and the quantity of ROS thanks to the action of flavonoids such as epigallocatechin gallate and gallic acid contained in green tea [109, 110]. Garlic increases NO, SOD, and GSH-Px activity and has an anti-inflammatory activity by reducing TNF-*α* expression [48, 111]. Synthetic antioxidants are N-acetyl-cysteine and propionyl-L-carnitine. N-Acetyl-cysteine is the intracellular precursor of glutathione, a substance with an excellent antioxidant activity furthermore minimizing oxidative stress in both endothelial cells and smooth muscle cells [112]. Sasaki et al. [113] showed that treatment with N-acetyl-L-cysteine or with pyrrolidine dithiocarbamate reduced TNF-*α*-induced MADCAM-1 expression. Propionyl-L-carnitine is an L-carnitine ester required in the transport of fatty acids for the production of *β* oxidation and adenosine triphosphate [114]. It has been proven to be a scavenger of superoxide, thus reducing oxidation stress in endothelial cells; indeed, Stasi et al. [115] demonstrated that propionyl-L-carnitine counteracted the increase of oxidative stress in the intestinal microvasculature of patients with UC. It also prevents NO decrease and therefore favors vasodilation, counteracting endothelial dysfunction, and it reduces NOX and ICAM-1 expression in experimental ischemia in rabbit limbs [115].

In literature, there are a large number of studies concerning the use of natural antioxidants in IBD, especially in animal models, which show how these substances with antioxidant properties can improve bowel damage both macroscopically and microscopically. In this regard, D'Argenio et al. [116] demonstrated the healthy effect of apple polyphenol extract in trinitrobenzensulphonic acid-induced colitis, an efficacy mediated by its effects on COX-2 and TNF-*α*. Binion et al. [117] demonstrated that curcumin reduced VCAM-1 expression. Zhang et al. demonstrated that *α*-lipoic acid, sulfhydryl compound, found in all plant and animal species, inhibits VCAM-1 expression by suppressing NF-*κ*B in human aortic endothelial cells. Sakthivel et al. [118] demonstrated the healthy effect of amentoflavone, which is a bioflavonoid active ingredient of the plant *Biophytum sensitivum* and of other plants, in an experimental colitis model since it inhibits iNOS and COX-2 expression [119].

## 3. Conclusions

A higher prevalence of classic cardiovascular risk factors is usually associated with a higher risk of cardiovascular events. However, this consideration cannot be applied to patients with IBD. Although patients with IBD have a lower prevalence of classic cardiovascular risk factors than in the general population, they have an increased risk of CVD. In patients with IBD, body mass index, lipid levels, diabetes, obesity, and hypertension are lower than in the general population [1, 4, 5, 120, 121]. In patients with IBD, there is an endothelial dysfunction that causes an increased arterial stiffness. There are no standardized therapies, and many studies in the literature evaluate how, reducing the endothelial dysfunction in patients with IBD, cardiovascular risk can be reduced. Endothelial dysfunction has inflammation and oxidative stress as its genesis. The effects of different therapies aimed at reducing these endothelial dysfunction mediators are not well known. Anti-TNF-*α* therapy appears to be associated with improvements in both endothelial function and arterial stiffness; however, further studies are needed to determine whether the improvements in arterial stiffness and endothelial function are associated with a decreased risk of cardiovascular events in subjects with IBD. With respect to natural or synthetic antioxidant substances, a large number of studies evaluate the effect on cardiovascular health. Furthermore, these studies demonstrate that vitamin E, vitamin C, goji berries, thymus extracts, rosemary, green tea, and garlic have a healthy effect on oxidative stress and inflammation, reducing them. Other substances, similar to antioxidants, were described, especially in models of experimental colitis, to be very effective in reducing macroscopic and microscopic damage, oxidative stress, and the most important mediators of inflammation. Consequently, we can suppose that in patients with IBD, these substances could be used as an adjunct to the traditional therapy, not only to improve the outcome of IBD but also to reduce cardiovascular risk. Further studies are needed to demonstrate the role of these substances.

## Figures and Tables

**Table 1:** Main studies on PWV for evaluation of arterial stiffness.

Authors (year)	Type of article	Studied people	Ref.
Laurent et al. (2006)	Consensus document	Healthy people and people with inflammatory diseases	[11]
Pietri et al. (2006)	Prospective study	Uncomplicated, never-treated Essential hypertension people	[12]
Yasmin et al. (2004)	Prospective study	Healthy people	[13]
Mäki-Petäjä et al. (2006)	Prospective study	Rheumatoid arthritis people and healthy people	[8]
Zanoli et al. (2012)	Prospective study	Inflammatory bowel disease people and healthy people	[15]
Akdoğan et al. (2013)	Prospective study	Ulcerative colitis people and healthy people	[16]
Korkmaz et al. (2014)	Prospective study	Inflammatory bowel disease people and healthy people	[17]
Aytac et al. (2015)	Prospective study	Inflammatory bowel disease people and healthy people	[18]
Zanoli et al. (2014)	Prospective study	Inflammatory bowel disease people and healthy people	[19]

**Table 2:** Mediators involved in endothelium dysfunction.

Author(s) (year)	Studied factors	Ref.
Garolla et al. (2009)	EPCs	[26]
Scaldaferri et al. (2011)	NO, endothelin 1, vWF, and CAM superfamily	[27]
Charo et al. (2006)	Integrins and chemokine receptors	[29]
Hatoum et al. (2003)	CAM superfamily and chemokines	[31]
Danese et al. (2011)	ICAM-1 and VCAM-1	[36]
Briskin et al. (1997) Burgio et al. (1995) Cromer et al. (2011)	MAdCAM-1, CD4, and *α*4*β*7 integrins	[34] [35] [36]
Wang et al. (2003) Pasceri et al. (2000) Venugopal et al. (2005) Clapp et al. (2005)	RCP	[37] [38] [39] [40]
Roifman et al. (2008) Danese et al. (2007) Danese et al. (2006) Kullo et al. (2005) Vita et al. (2004)	IL-1, TNF-*α*, NO, VEGF, CD40-CD40-ligand, and IL-6	[41] [42] [43] [44] [45]
Floege et al. (2004)	IL-1, TNF-*α*, ROS, matrix metalloproteinases, serine proteinases	[46]
Koutroubakis et al. (2006)	Inflammatory cells (macrophages, lymphocytes, mast cells, and fibroblasts),VEGF, and TNF-*α*	[47]
Horowitz et al. (2007)	NO	[49]
Steyers et al. (2014)	TNF-*α* and NO	[22]
Sibal et al. (2010) Boger et al. (2009)	ADMA	[51] [52]
Kleinbongard et al. (2010) Picchi et al. (2006)	TNF-*α*	[53] [54]
Kalinowski et al. (2004)	NADH/NADPH	[55]
Kundu et al. (2012) Wolin (2000)	ROS, NF-*κ*B, and CAM superfamily	[56] [57]
Biniecka et al. (2011)	NF-*κ*B, NOX, and ROS	[58]
Lancellotti et al. (2010)	vWF	[59]
Ciccone et al. (2015) Desreumaux et al. 1(999)	PAI-1, t-PA, and u-PA	[60] [61]

EPCs: endothelial precursor cells; NO: nitric oxide; vWF: von Willebrand factor; CAM: cell adhesion molecule; ICAM-1: intercellular adhesion molecule; VCAM-1: vascular adhesion molecule; MAdCAM-1: mucosal addressin cell adhesion molecule-1; RCP: reactive C protein; IL: interleukin; TNF: tumor necrosis factor; VEGF: vascular endothelial growth factor; ROS: reactive oxygen species; ADMA: asymmetric dimethylarginine; NAD: nicotinamide adenine dinucleotide; NADP: nicotinamide adenine dinucleotide phosphate; NF-*κ*B: nuclear factor kB; PAI: plasminogen activator inhibitor; t-PA: tissue-type plasminogen activator; u-PA: urokinase-type plasminogen activator.

**Table 3:** Most popular parameters for diagnosis of endothelium dysfunction.

*Biochemical parameters*
Intercellular adhesion molecule-1 (CAM-1)
Selectins P and E
Vascular adhesion molecule-1 (VCAM-1)
Vascular endothelial growth factor (VEGF)
von Willebrand factor (vWF)
Tissue plasminogen activator (t-PA)
Thrombomodulin
Plasminogen activator inhibitor (PAI-1)
Asymmetric dimethylarginine (ADMA)
Disintegrin and metalloproteinase with thrombospondin motif-13 (ADAMTS13)
Angiopoietin-1
Angiopoietin-2
*Physical parameters*
Flow-mediated dilatation (FMD)
Carotid intima-media thickness (c-IMT)
Pulse wave velocity (PWV)
Pulse arterial tonometry (PAT)

**Table 4:** Association between cardiovascular diseases and inflammatory bowel diseases.

Authors (year)	Association	Ref.
Yarur et al. (2011) Ciccone et al. (2015) Rungoe et al. (2013) Fumery et al. (2014) Singh et al. (2014)	Yes	[5] [60] [75] [88] [89]
Dorn et al. (2007) Jess et al. (2007) Bewtra et al. (2013) Ruisi et al. (2015)	No	[6] [76] [77] [122]

**Table 5:** Natural antioxidant compounds for prevention of vascular damage.

Author (year)	Compound	Ref
Bielli et al. (2015) Tran et al. (1990) Brigelius-Flohé et al. (2013)	Vitamin E	[48] [96] [97]
Bielli et al. (2015) Armour et al. (2001) May et al. (2013) Heitzer et al. (1996)	Vitamin C	[48] [98] [99] [100]
Bielli et al. (2015) Amagase et al. (2009) Li et al. (2007)	Goji berries	[48] [101] [102]
Martins et al. (2015) Nickavar et al. (2012)	Thymus extracts	[107] [108]
Bielli et al. (2015)	Rosemary	[48]
Murase et al. (2006) Lu et al. (2012)	Green tea	[109] [110]
Bielli et al. (2015) Kim et al. (2001)	Garlic	[48] [111]

## References

[1] Zanoli L., Rastelli S., Inserra G., Castellino P. (2015). Arterial structure and function in inflammatory bowel disease. *World Journal of Gastroenterology*.

[2] Tian T., Wang Z., Zhang J. (2017). Pathomechanisms of oxidative stress in inflammatory bowel disease and potential antioxidant therapies. *Oxidative Medicine and Cellular Longevity*.

[3] Corridoni D., Arseneau K. O., Cominelli F. (2014). Inflammatory bowel disease. *Immunology Letters*.

[4] Levy E., Rizwan Y., Thibault L. (2000). Altered lipid profile, lipoprotein composition, and oxidant and antioxidant status in pediatric Crohn disease. *The American Journal of Clinical Nutrition*.

[5] Yarur A. J., Deshpande A. R., Pechman D. M., Tamariz L., Abreu M. T., Sussman D. A. (2011). Inflammatory bowel disease is associated with an increased incidence of cardiovascular events. *The American Journal of Gastroenterology*.

[6] Dorn S. D., Sandler R. S. (2007). Inflammatory bowel disease is not a risk factor for cardiovascular disease mortality: results from a systematic review and meta-analysis. *The American Journal of Gastroenterology*.

[7] Booth A. D., Wallace S., McEniery C. M. (2004). Inflammation and arterial stiffness in systemic vasculitis: a model of vascular inflammation. *Arthritis and Rheumatism*.

[8] Mäki-Petäjä K. M., Hall F. C., Booth A. D. (2006). Rheumatoid arthritis is associated with increased aortic pulsewave velocity, which is reduced by anti-tumor necrosis factor-alpha therapy. *Circulation*.

[9] Roman M. J., Devereux R. B., Schwartz J. E. (2005). Arterial stiffness in chronic inflammatory diseases. *Hypertension*.

[10] Mancia G., Fagard R., Narkiewicz K. (2013). 2013 Practice guidelines for the management of arterial hypertension of the European Society of Hypertension (ESH) and the European Society of Cardiology (ESC). *Journal of Hypertension*.

[11] Laurent S., Cockcroft J., Van Bortel L. (2006). Expert consensus document on arterial stiffness: methodological issues and clinical applications. *European Heart Journal*.

[12] Pietri P., Vyssoulis G., Vlachopoulos C. (2006). Relationship between low-grade inflammation and arterial stiffness in patients with essential hypertension. *Journal of Hypertension*.

[13] Yasmin Y., McEniery C., Wallace S., Mackenzie I., Cockcroft J., Wilkinson I. (2004). C-reactive protein is associated with arterial stiffness in apparently healthy individuals. *Arteriosclerosis, Thrombosis, and Vascular Biology*.

[14] McDonnell S., Morgan M., Lynch C. (1999). Role of matrix metalloproteinases in normal and disease processes. *Biochemical Society Transactions*.

[15] Zanoli L., Cannavò M., Rastelli S. (2012). Arterial stiffness is increased in patients with inflammatory bowel disease. *Journal of Hypertension*.

[16] Akdoğan R. A., Durakoğlugil M. E., Kocaman S. A. (2013). Increased pulse wave velocity and carotid intima-media thickness in patients with ulcerative colitis. *Digestive Diseases and Sciences*.

[17] Korkmaz H., Sahin F., Ipekci S. H., Temel T., Kebapcilar L. (2014). Increased pulse wave velocity and relationship with inflammation, insulin, and insulin resistance in inflammatory bowel disease. *European Journal of Gastroenterology & Hepatology*.

[18] Aytac E., Buyuktas D., Baysal B. (2015). Visual evoked potentials and pulse wave velocity in inflammatory bowel disease. *The Turkish Journal of Gastroenterology*.

[19] Zanoli L., Rastelli S., Inserra G. (2014). Increased arterial stiffness in inflammatory bowel diseases is dependent upon inflammation and reduced by immunomodulatory drugs. *Atherosclerosis*.

[20] Cibor D., Domagala-Rodacka R., Rodacki T., Jurczyszyn A., Mach T., Owczarek D. (2016). Endothelial dysfunction in inflammatory bowel diseases: pathogenesis, assessment and implications. *World Journal of Gastroenterology*.

[21] Deanfield J., Donald A., Ferri C. (2005). Endothelial function and dysfunction. Part I: methodological issues for assessment in the different vascular beds. *Journal of Hypertension*.

[22] Steyers C., Miller F. (2014). Endothelial dysfunction in chronic inflammatory diseases. *International Journal of Molecular Sciences*.

[23] Ciccone M. M., Bilianou E., Balbarini A. (2013). Task force on. *Journal of Cardiovascular Medicine*.

[24] Lerman A., Zeiher A. M. (2005). Endothelial function: cardiac events. *Circulation*.

[25] Schinzari F., Armuzzi A., De Pascalis B. (2007). Tumor necrosis factor-*α* antagonism improves endothelial dysfunction in patients with Crohn’s disease. *Clinical Pharmacology & Therapeutics*.

[26] Garolla A., D’Incà R., Checchin D. (2009). Reduced endothelial progenitor cell number and function in inflammatory bowel disease: a possible link to the pathogenesis. *The American Journal of Gastroenterology*.

[27] Scaldaferri F., Lancellotti S., Pizzoferrato M., De Cristofaro R. (2011). Haemostatic system in inflammatory bowel diseases: new players in gut inflammation. *World Journal of Gastroenterology*.

[28] Deban L., Correale C., Vetrano S., Malesci A., Danese S. (2008). Multiple pathogenic roles of microvasculature in inflammatory bowel disease: a jack of all trades. *The American Journal of Pathology*.

[29] Charo I. F., Ransohoff R. M. (2006). The many roles of chemokines and chemokine receptors in inflammation. *The New England Journal of Medicine*.

[30] D’Alessio S., Tacconi C., Fiocchi C., Danese S. (2013). Advances in therapeutic interventions targeting the vascular and lymphatic endothelium in inflammatory bowel disease. *Current Opinion in Gastroenterology*.

[31] Hatoum O. A., Miura H., Binion D. G. (2003). The vascular contribution in the pathogenesis of inflammatory bowel disease. *American Journal of Physiology. Heart and Circulatory Physiology*.

[32] Danese S., Semeraro S., Marini M. (2005). Adhesion molecules in inflammatory bowel disease: therapeutic implications for gut inflammation. *Digestive and Liver Disease*.

[33] Danese S. (2011). Role of the vascular and lymphatic endothelium in the pathogenesis of inflammatory bowel disease: ‘brothers in arms’. *Gut*.

[34] Briskin M., Winsor-Hines D., Shyjan A. (1997). Human mucosal addressin cell adhesion molecule-1 is preferentially expressed in intestinal tract and associated lymphoid tissue. *The American Journal of Pathology*.

[35] Lelio Burgio V., Fais S., Boirivant M., Perrone A., Pallone F. (1995). Peripheral monocyte and naive T-cell recruitment and activation in Crohn’s disease. *Gastroenterology*.

[36] Cromer W. E., Mathis J. M., Granger D. N., Chaitanya G. V., Alexander J. S. (2011). Role of the endothelium in inflammatory bowel diseases. *World Journal of Gastroenterology*.

[37] Wang C. H., Li S. H., Weisel R. D. (2003). C-reactive protein upregulates angiotensin type 1 receptors in vascular smooth muscle. *Circulation*.

[38] Pasceri V., Willerson J. T., Yeh E. T. H. (2000). Direct proinflammatory effect of C-reactive protein on human endothelial cells. *Circulation*.

[39] Venugopal S. K., Devaraj S., Jialal I. (2005). Macrophage conditioned medium induces the expression of C-reactive protein in human aortic endothelial cells. *The American Journal of Pathology*.

[40] Clapp B. R., Hirschfield G. M., Storry C. (2005). Inflammation and endothelial function: direct vascular effects of human C-reactive protein on nitric oxide bioavailability. *Circulation*.

[41] Roifman I., Sun Y. C., Fedwick J. P. (2009). Evidence of endothelial dysfunction in patients with inflammatory bowel disease. *Clinical Gastroenterology and Hepatology*.

[42] Danese S., Scaldaferri F., Vetrano S. (2007). Critical role of the CD40 CD40-ligand pathway in regulating mucosal inflammation-driven angiogenesis in inflammatory bowel disease. *Gut*.

[43] Liu Y. H., Ding Y., Gao C. C., Li L. S., Wang Y. X., Xu J. D. (2018). Functional macrophages and gastrointestinal disorders. *World Journal of Gastroenterology*.

[44] Kullo I., Seward J., Bailey K. (2005). C-reactive protein is related to arterial wave reflection and stiffness in asymptomatic subjects from the community. *American Journal of Hypertension*.

[45] Vita J. A., Keaney J. F., Larson M. G. (2004). Brachial artery vasodilator function and systemic inflammation in the Framingham Offspring Study. *Circulation*.

[46] Floege J., Ketteler M. (2004). Vascular calcification in patients with endstage renal disease. *Nephrology, Dialysis, Transplantation*.

[47] Koutroubakis I. E., Tsiolakidou G., Karmiris K., Kouroumalis E. A. (2006). Role of angiogenesis in inflammatory bowel disease. *Inflammatory Bowel Diseases*.

[48] Bielli A., Scioli M. G., Mazzaglia D., Doldo E., Orlandi A. (2015). Antioxidants and vascular health. *Life Sciences*.

[49] Horowitz S., Binion D. G., Nelson V. M. (2007). Increased arginase activity and endothelial dysfunction in human inflammatory bowel disease. *American Journal of Physiology-Gastrointestinal and Liver Physiology*.

[50] Magro F., Soares J. B., Fernandes D. (2014). Venous thrombosis and prothrombotic factors in inflammatory bowel disease. *World Journal of Gastroenterology*.

[51] Sibal L., C Agarwal S., D Home P., H Boger R. (2010). The role of asymmetric dimethylarginine [ADMA] in endothelial dysfunction and cardiovascular disease. *Current Cardiology Reviews*.

[52] Boger R. H., Maas R., Schulze F., Schwedhelm E. (2009). Asymmetric dimethylarginine [ADMA] as a prospective marker of cardiovascular disease and mortality--an update on patient populations with a wide range of cardiovascular risk. *Pharmacological Research*.

[53] Kleinbongard P., Heusch G., Schulz R. (2010). TNFalpha in atherosclerosis, myocardial ischemia/reperfusion and heart failure. *Pharmacology & Therapeutics*.

[54] Picchi A., Gao X., Belmadani S. (2006). Tumor necrosis factor-alpha induces endothelial dysfunction in the prediabetic metabolic syndrome. *Circulation Research*.

[55] Kalinowski L., Malinski T. (2004). Endothelial NADH/NADPH-dependent enzymatic sources of superoxide production: relationship to endothelial dysfunction. *Acta Biochimica Polonica*.

[56] Kundu S., Ghosh P., Datta S., Ghosh A., Chattopadhyay S., Chatterjee M. (2012). Oxidative stress as a potential biomarker for determining disease activity in patients with rheumatoid arthritis. *Free Radical Research*.

[57] Wolin M. S. (2000). Interactions of oxidants with vascular signaling systems. *Arteriosclerosis, Thrombosis, and Vascular Biology*.

[58] Biniecka M., Kennedy A., Ng C. T. (2011). Successful tumour necrosis factor [TNF] blocking therapy suppresses oxidative stress and hypoxia-induced mitochondrial mutagenesis in inflammatory arthritis. *Arthritis Research & Therapy*.

[59] Lancellotti S., De Filippis V., Pozzi N. (2010). Formation of methionine sulfoxide by peroxynitrite at position 1606 of von Willebrand factor inhibits its cleavage by ADAMTS-13: a new prothrombotic mechanism in diseases associated with oxidative stress. *Free Radical Biology & Medicine*.

[60] Ciccone M. M., Principi M., Ierardi E. (2015). Inflammatory bowel disease, liver diseases and endothelial function: is there a linkage?. *Journal of Cardiovascular Medicine*.

[61] Desreumaux P., Huet G., Zerimech F. (1999). Acute inflammatory intestinal vascular lesions and in situ abnormalities of the plasminogen activation system in Crohn’s disease. *European Journal of Gastroenterology & Hepatology*.

[62] Principi M., Mastrolonardo M., Scicchitano P. (2013). Endothelial function and cardiovascular risk in active inflammatory bowel diseases. *Journal of Crohn’s and Colitis*.

[63] Theocharidou E., Gossios T. D., Griva T. (2013). Is there an association between inflammatory bowel diseases and carotid intima-media thickness? Preliminary data. *Angiology*.

[64] van Leuven S. I., Hezemans R., Levels J. H. (2007). Enhanced atherogenesis and altered high density lipoprotein in patients with Crohn’s disease. *Journal of Lipid Research*.

[65] Papa A., Santoliquido A., Danese S. (2005). Increased carotid intima–media thickness in patients with inflammatory bowel disease. *Alimentary Pharmacology & Therapeutics*.

[66] Dagli N., Poyrazoglu O. K., Ferda Dagli A. (2009). Is inflammatory bowel disease a risk factor for early atherosclerosis?. *Angiology*.

[67] Maharshak N., Arbel Y., Bornstein N. M. (2007). Inflammatory bowel disease is not associated with increased intimal media thickening. *The American Journal of Gastroenterology*.

[68] Broide E., Schopan A., Zaretsky M., Kimchi N. A., Shapiro M., Scapa E. (2011). Intima media thickness of the common carotid artery is not significantly higher in Crohn’s disease patients compared to healthy population. *Digestive Diseases and Sciences*.

[69] Di Sabatino A., Ciccocioppo R., Armellini E. (2004). Serum bFGF and VEGF correlate respectively with bowel wall thickness and intramural blood flow in Crohn’s disease. *Inflammatory Bowel Diseases*.

[70] Song W. B., Lv Y. H., Zhang Z. S. (2009). Soluble intercellular adhesion molecule-1, D-lactate and diamine oxidase in patients with inflammatory bowel disease. *World Journal of Gastroenterology*.

[71] Magro F., Araujo F., Pereira P., Meireles E., Diniz-Ribeiro M., Velosom F. T. (2004). Soluble selectins, sICAM, sVCAM, and angiogenic proteins in different activity groups of patients with inflammatory bowel disease. *Digestive Diseases and Sciences*.

[72] Murdaca G., Colombo B. M., Cagnati P., Gulli R., Spano F., Puppo F. (2012). Endothelial dysfunction in rheumatic autoimmune diseases. *Atherosclerosis*.

[73] Prati C., Demougeot C., Guillot X., Godfrin-Valnet M., Wendling D. (2014). Endothelial dysfunction in joint disease. *Joint, Bone, Spine*.

[74] Davignon J., Ganz P. (2004). Role of endothelial dysfunction in atherosclerosis. *Circulation*.

[75] Rungoe C., Basit S., Ranthe M. F., Wohlfahrt J., Langholz E., Jess T. (2013). Risk of ischaemic heart disease in patients with inflammatory bowel disease: a nationwide Danish cohort study. *Gut*.

[76] Jess T., Gamborg M., Munkholm P., Sørensen T. I. A. (2007). Overall and causespecific mortality in ulcerative colitis: meta-analysis of population based inception cohort studies. *The American Journal of Gastroenterology*.

[77] Bewtra M., Kaiser L. M., TenHave T., Lewis J. D. (2013). Crohn’s disease and ulcerative colitis are associated with elevated standardized mortality ratios: a meta-analysis. *Inflammatory Bowel Diseases*.

[78] Ozturk K., Guler A. K., Cakir M. (2015). Pulse wave velocity, intima media thickness, and flow-mediated dilatation in patients with normotensive normoglycemic inflammatory bowel disease. *Inflammatory Bowel Diseases*.

[79] Ciccone M. M., Miniello V., Marchioli R. (2011). Morphological and functional vascular changes induced by childhood obesity. *European Journal of Cardiovascular Prevention and Rehabilitation*.

[80] Caliskan Z., Keles N., Gokturk H. S. (2016). Is activation in inflammatory bowel diseases associated with further impairment of coronary microcirculation?. *International Journal of Cardiology*.

[81] Hatoum O. A., Binion D. G. (2005). The vasculature and inflammatory bowel disease: contribution to pathogenesis and clinical pathology. *Inflammatory Bowel Diseases*.

[82] Kaptoge S., Seshasai S. R. K., Gao P. (2014). Inflammatory cytokines and risk of coronary heart disease: new prospective study and updated metaanalysis. *European Heart Journal*.

[83] Van Doornum S., McColl G., Jenkins A., Green D. J., Wicks I. P. (2003). Screening for atherosclerosis in patients with rheumatoid arthritis: comparison of two in vivo tests of vascular function. *Arthritis and Rheumatism*.

[84] Ciccone M. M., De Pergola G., Porcelli M. T. (2010). Increased carotid IMT in overweight and obese women affected by Hashimoto’s thyroiditis: an adiposity and autoimmune linkage?. *BMC Cardiovascular Disorders*.

[85] Kristensen S. L., Ahlehoff O., Lindhardsen J. (2014). Prognosis after first-time myocardial infarction in patients with inflammatory bowel disease according to disease activity: nationwide cohort study. *Circulation Cardiovascular Quality and Outcomes*.

[86] Principi M., Montenegro L., Losurdo G. (2015). Endothelial function and cardiovascular risk in patients with inflammatory bowel disease in remission phase. *Scandinavian Journal of Gastroenterology*.

[87] Ruemmele F. M., Veres G., Kolho K. L. (2014). Consensus guidelines of ECCO/ESPGHAN on the medical management of pediatric Crohn’s disease. *Journal of Crohn’s & Colitis*.

[88] Singh S., Singh H., Loftus E. V., Pardi D. S. (2014). Risk of cerebrovascular accidents and ischemic heart disease in patients with inflammatory bowel disease: a systematic review and meta-analysis. *Clinical Gastroenterology and Hepatology*.

[89] Fumery M., Xiaocang C., Dauchet L., Gower-Rousseau C., Peyrin-Biroulet L., Colombel J. F. (2014). Thromboembolic events and cardiovascular mortality in inflammatory bowel diseases: a meta-analysis of observational studies. *Journal of Crohn’s & Colitis*.

[90] Szpak D., Grochowalski A., Chrząszcz R., Florek E., Jawień W., Undas A. (2013). Tobacco smoke exposure and endothelial dysfunction in patients with advanced coronary artery disease. *Polish Archives of Internal Medicine*.

[91] Ray M., Autieri M. V. (2017). Regulation of pro- and anti-atherogenic cytokines. *Cytokine*.

[92] Wang P., Ba Z. F., Chaudry I. H. (1994). Administration of tumor necrosis factor-alpha in vivo depresses endothelium-dependent relaxation. *American Journal of Physiology-Heart and Circulatory Physiology*.

[93] Kotani K., Miyamoto M., Ando H. (2017). The effect of treatments for rheumatoid arthritis on endothelial dysfunction evaluated by flow-mediated vasodilation in patients with rheumatoid arthritis. *Current Vascular Pharmacology*.

[94] Danese S., Sans M., Scaldaferri F. (2006). TNF-alpha blockade down-regulates the CD40/CD40L pathway in the mucosal microcirculation: a novel anti-inflammatory mechanism of infliximab in Crohn’s disease. *Journal of Immunology*.

[95] Lü J. M., Lin P. H., Yao Q., Chen C. (2009). Chemical and molecular mechanisms of antioxidants: experimental approaches and model systems. *Journal of Cellular and Molecular Medicine*.

[96] Tran K., Chan A. C. (1990). R,R,R-*α*-tocopherol potentiates prostacyclin release in human endothelial cells. Evidence for structural specificity of the tocopherol molecule. *Biochimica et Biophysica Acta (BBA) - Lipids and Lipid Metabolism*.

[97] Brigelius-Flohé R., Traber M. G. (1999). Vitamin E: function and metabolism. *The FASEB Journal*.

[98] Armour J., Tyml K., Lidington D., Wilson J. X. (2001). Ascorbate prevents microvascular dysfunction in the skeletal muscle of the septic rat. *Journal of Applied Physiology*.

[99] May J. M., Harrison F. E. (2013). Role of vitamin C in the function of the vascular endothelium. *Antioxidants & Redox Signaling*.

[100] Heitzer T., Just H., Münzel T. (1996). Antioxidant vitamin C improves endothelial dysfunction in chronic smokers. *Circulation*.

[101] Amagase H., Sun B., Borek C. (2009). Lycium barbarum [goji] juice improves in vivo antioxidant biomarkers in serum of healthy adults. *Nutrition Research*.

[102] Li X. M., Ma Y. L., Liu X. J. (2007). Effect of the Lycium barbarum polysaccharides on age-related oxidative stress in aged mice. *Journal of Ethnopharmacology*.

[103] Keshavarzian A., Morgan G., Sedghi S., Gordon J. H., Doria M. (1990). Role of reactive oxygen metabolites in experimental colitis. *Gut*.

[104] Xia B., Deng C. S., Chen D. J., Zhou Y., Xiao J. Q. (1996). Role of copper zinc superoxide dismutase in the short-term treatment of acetic acid-induced colitis in rats. *Acta Gastroenterologica Latinoamericana*.

[105] Seguí J., Gil F., Gironella M. (2005). Down-regulation of endothelial adhesion molecules and leukocyte adhesion by treatment with superoxide dismutase is beneficial in chronic immune experimental colitis. *Inflammatory Bowel Diseases*.

[106] Seguí J., Gironella M., Sans M. (2004). Superoxide dismutase ameliorates TNBS-induced colitis by reducing oxidative stress, adhesion molecule expression, and leukocyte recruitment into the inflamed intestine. *Journal of Leukocyte Biology*.

[107] Martins N., Barros L., Santos-Buelga C., Silva S., Henriques M., Ferreira I. C. F. R. (2015). Decoction, infusion and hydroalcoholic extract of cultivated thyme: antioxidant and antibacterial activities, and phenolic characterisation. *Food Chemistry*.

[108] Nickavar B., Esbati N. (2012). Evaluation of the antioxidant capacity and phenolic content of three thymus species. *Journal of Acupuncture and Meridian Studies*.

[109] Murase T., Haramizu S., Shimotoyodome A., Tokimitsu I., Hase T. (2006). Green tea extract improves running endurance in mice by stimulating lipid utilization during exercise. *American Journal of Physiology Regulatory, Integrative and Comparative Physiology*.

[110] Lu Q.-Y., Jin Y., Mao J. T. (2012). Green tea inhibits cycolooxygenase-2 in non-small cell lung cancer cells through the induction of annexin-1. *Biochemical and Biophysical Research Communications*.

[111] Kim K. M., Chun S. B., Koo M. S. (2001). Differential regulation of NO availability from macrophages and endothelial cells by the garlic component S-allyl cysteine. *Free Radical Biology & Medicine*.

[112] Rodrigues S. D., França K. C., Dallin F. T. (2015). N-acetylcysteine as a potential strategy to attenuate the oxidative stress induced by uremic serum in the vascular system. *Life Sciences*.

[113] Sasaki M., Ostanin D., Elrod J. W. (2003). TNF-*α*-induced endothelial cell adhesion molecule expression is cytochromeP-450 monooxygenase dependent. *American Journal of Physiology Cell Physiology*.

[114] Ribas G. S., Vargas C. R., Wajner M. (2014). L-Carnitine supplementation as a potential antioxidant therapy for inherited neurometabolic disorders. *Gene*.

[115] Stasi M. A., Scioli M. G., Arcuri G. (2010). Propionyl-L-carnitine improves postischemic blood flow recovery and arteriogenetic revascularization and reduces endothelial NADPH-oxidase 4-mediated superoxide production. *Arteriosclerosis, Thrombosis, and Vascular Biology*.

[116] D’Argenio G., Mazzone G., Tuccillo C. (2012). Apple polyphenols extract (APE) improves colon damage in a rat model of colitis. *Digestive and Liver Disease*.

[117] Binion D. G., Heidemann J., Li M. S., Nelson V. M., Otterson M. F., Rafiee P. (2009). Vascular cell adhesion molecule-1 expression in human intestinal microvascular endothelial cells is regulated by PI 3-kinase/Akt/MAPK/NF-kappaB: inhibitory role of curcumin. *American Journal of Physiology-Gastrointestinal and Liver Physiology*.

[118] Zhang W. J., Frei B. (2001). Alpha-lipoic acid inhibits TNF-alpha-induced NF-kappaB activation and adhesion molecule expression in human aortic endothelial cells. *The FASEB Journal*.

[119] Sakthivel K. M., Guruvayoorappan C. (2013). Amentoflavone inhibits iNOS, COX-2 expression and modulates cytokine profile, NF-*κ*B signal transduction pathways in rats with ulcerative colitis. *International Immunopharmacology*.

[120] Geerling B. J., Badart-Smook A., Stockbrügger R. W., Brummer R. J. (2000). Comprehensive nutritional status in recently diagnosed patients with inflammatory bowel disease compared with population controls. *European Journal of Clinical Nutrition*.

[121] Jahnsen J., Falch J. A., Mowinckel P., Aadland E. (2003). Body composition in patients with inflammatory bowel disease: a population-based study. *The American Journal of Gastroenterology*.

[122] Ruisi P., Makaryus J. N., Ruisi M., Makaryus A. N. (2015). Inflammatory bowel disease as a risk factor for premature coronary artery disease. *Journal of Clinical Medicine Research*.

